# Exploration of Fatalism and Religiosity by Gender and Varying Levels of Engagement Among Mexican-American Adults of a Type 2 Diabetes Management Program

**DOI:** 10.3389/fpubh.2021.652202

**Published:** 2021-09-27

**Authors:** Cindy Lynn Salazar-Collier, Belinda M. Reininger, Anna V. Wilkinson, Steven H. Kelder

**Affiliations:** ^1^Department of Public Health, College of Nursing and Health Sciences, Texas A&M International University, Laredo, TX, United States; ^2^Department of Health Promotion & Behavioral Sciences, School of Public Health, The University of Texas Health Science Center at Houston, Brownsville, TX, United States; ^3^Deaprtment of Epidemiology, Human Genetics & Environmental Sciences, School of Public Health, The University of Texas Health Science at Houston, Austin, TX, United States

**Keywords:** Fatalism, Religiosity, Diabetes management, Hispanic health, Mexican-American border region

## Abstract

**Objectives:** Purpose of study is to explore the roles religiosity and fatalistic beliefs play in diabetes management among newly, currently, and long-term enrolled Mexican-American participants in a Type 2 diabetes mellitus (T2DM) chronic care management program.

**Methods:** In 2017, study participants (*n* = 15) completed a semi-structured interview in their preferred language (English or Spanish). Sample was stratified by amount of time individual had been enrolled as a participant of the Salud y Vida program: newly, currently, or long-term. Interviews assessed religious beliefs, beliefs concerning the cause(s) of diabetes, perceived relationship between religiosity and fatalistic beliefs with T2DM management, and the appropriateness of discussing such topics with a health professional. Interview responses were analyzed using ATLAS.ti 8.

**Results:** Themes identified included: perceived autonomy over diabetes prognosis, motivators for self-care, discussions of personal beliefs in the healthcare setting, and the church's role in diabetes management.

**Conclusions:** Among this sample, religiosity and religious fatalism played a complex role in coping with and managing diabetes. Long-term enrolled and male participants expressed beliefs of divine control over health, and a connection between religiosity and health behavior. Long-term enrolled participants felt religious and fatalistic beliefs may be suitable and beneficial to discuss in the healthcare setting.

## Introduction

Over one in 10 (11.8%) Hispanic adults in the United States are estimated to have Type 2 diabetes mellitus (T2DM) (1). Estimates among Mexican Americans range from 13.3 to 18.1%, compared to 7.1% of non-Hispanic whites (2). Hispanics not only have a high prevalence of T2DM, they also have the highest rates of uninsured adults (32%) when compared to other racial and ethnic groups (3). Moreover, Hispanic patients with T2DM have a lower prevalence of meeting American Diabetes Association standards for blood glucose management (HbA1c ≤ 7.0%) when compared to non-Hispanic whites (24.4 vs. 38.0%) (4). Socioeconomic factors, health literacy, physician-patient relationships, personal factors and beliefs all play a role in T2DM management (5, 6).

Fatalism, which may play a role in diabetes management, is the belief that every event and circumstance is predetermined, and an individual is powerless in altering the progression of these events (7–9). Fatalism includes the concepts of luck, fate, and destiny. Fatalism is strongly related to the concept of locus of control introduced by Rotter, which posits that although reinforcement, rewards, and gratification are central in developing skills and behaviors, the acquirement of these behaviors is contingent on the manner in which the individual perceived this reward (10). Qualitative studies assessing fatalistic beliefs and their effect on health behaviors among Hispanic adults have provided mixed results (8, 9). One such study found fatalistic beliefs to be a barrier to adopting positive self-care behaviors, namely dietary behaviors among Hispanic men, while another did not find fatalism to be a barrier to diabetes self-management (8, 9). The topic of fatalism is of increasing interest in Latino health specifically to determine whether fatalism truly has an influence on an individual's choice of whether or not to engage in preventative health behaviors (11). Literature on fatalism, to date demonstrates mixed results among Latino populations, particularly in the area of cancer screening. Limited research has been conducted on this construct in relation to prevention and management of diabetes. A methodological issue also is present in the research on fatalism in cancer screening behaviors which is that many studies use a scale that measures distinct fatalism constructs rather than a comprehensive set of scales, which limits and may differentially affect the understanding of the screening behaviors. Ultimately the methodological issue may attenuate the effect of fatalism and inhibit the understanding of the positive or negative effects the fatalistic beliefs have on coping or managing illness. The present study aims to determine what constructs of fatalism, if any, play a role in diabetes management in an effort to better inform fatalism measurement among Latinos in the context of diabetes management.

Religiosity, a related yet distinct construct, is the belief in a deity who exercises control over all human life (12). In past years, researchers have described religiosity broadly including an individual's religious beliefs, relationships within the church, and devout practices. However, recent definitions of religiosity have focused on religious practices, and do not account for the element of spirituality or the interplay between religion and fatalism (13). Religion is a central part of Hispanic culture with 55% of Hispanics identifying as Roman Catholic, 22% identifying as Protestant, and only 18% identifying as non-believers (14). Religion plays a complex role in an individual's life and may promote a healthier lifestyle through its prescriptions of healthy behaviors and proscriptions of unhealthy behaviors (15). A qualitative study among 43 Mexican-Americans with T2DM found that a belief in God indirectly helped by lowering stress and anxiety (16). Similarly, among Hispanic migrant workers with T2DM, prayer was used as a source of comfort by praying for their families' and their own health, and reciting prayers specifically for their diabetes (17). Among a sample of 104 Hispanics with T2DM, 55% sought help from their priest to control their diabetes (18).

Given the impact that fatalism and religiosity have in determining health outcomes and disease management behaviors, in both indirect and direct manners, among Hispanic populations, the present study aims to explore the roles both of these beliefs play in diabetes care management among newly, currently, and long-term enrolled Mexican-American participants of a T2DM chronic care management program. Previous qualitative studies assessing the effects of fatalistic beliefs on diabetes management have provided inconsistent results (8, 9). Those assessing the effect of religiosity on diabetes management have explored this relationship without assessing the influence of fatalism. Although these are distinct constructs, they are related and may both play a role in diabetes behaviors (16, 17).

## Methods

### Design

Qualitative methods were utilized, and semi-structured interviews were conducted with each participant in their preferred language (English or Spanish) (19–21). Questions addressed participants' personal religious beliefs, lifetime religiosity, perceived effect of religiosity and fatalistic beliefs on diabetes management; diabetes etiology, use of religious and folk practices for T2DM management, and appropriateness of discussing such topics with health care practitioners. Interview questions can be found in Table 1. Egede's work exploring fatalism among an African-American population, a qualitative study among Iranian patients with T2DM looking at religiosity and the empowerment process, and semi-structured focus group questions utilized with a Hispanic population in the context of cancer fatalism were utilized to inform the development of this guide (19–21). Questions were tailored to a Hispanic population to ensure they were at the appropriate literacy level, and addressed culturally relevant elements of religiosity and fatalism, such as folk medicine beliefs and practices. The present study was reviewed and approved by the University of Texas Health Science Center at Houston School of Public Health Institutional Review Board, IRB approval number HSC-SPH-15-0600.

**Table 1:** Interview guide.

**Introduction**
1. Please describe things that are important in your daily life. For example, this can include your family, work, your church, or a hobby.
**Religiosity**
2. Please describe any importance, if at all, that religion and/or your faith has in your daily life?Probe: In what ways do you engage in your religious beliefs in your daily life? For example, attending church, prayer, confessions, volunteering with your church, or any other church activity.
3. How has the role religion plays in your life changed from when you were a child to when you were an adult? If the role that religion played has changed please describe how it has changed.Probe: Describe differences in religious practices from when you were a child and now as an adult. For example, religious denomination, church attendance, prayer, confessions, or any other church activity.Probe: What prompted these changes? (if applicable).
4. For some people their religious beliefs could influence their choices about health, which could include what they eat, drink, or choosing to take certain medications. For other people it does not influence them at all. In what way, if at all, would you say that your religious beliefs influence your Type 2 diabetes management?Probe: Describe any religious messages or themes that encourage healthy lifestyles and any influence those messages have on your health choices.
**Fatalism**
5. Fate is described as the occurrence of events beyond a person's control, and is regarded as determined by a supernatural higher power, which may or may not include religious beliefs. Describe your beliefs in regard to fate. Describe your beliefs about fate in relation with Type 2 diabetes?Probe: Who or what is responsible for a person's fate? Is there anything someone can do to change their fate?
**Type 2 Diabetes etiology**
6. Some people believe there are different ways that someone could get diabetes. Some beliefs include an intense fear known as “susto,” a curse from someone else, a person's diet, or that it was inherited. I am very interested in these broad range of the beliefs. Please describe your beliefs as to how a person develops Type 2 diabetes. Describe your beliefs in regard to the role God plays in a person developing diabetes and the outcomes of the disease.
**Diabetes Management behaviors**
7. Describe any religious practices (such as prayer) or cultural practices (such as herbal medicine, ointments, teas or pills) that you use as a method to help with your Type 2 diabetes management.Probe: Describe how you think prayer can or cannot help a person manage their diabetes.
8. Healthy behaviors as you know include taking your medication, eating healthy, checking your blood sugar every day, attending your doctor's visits, and engaging in physical activity. Describe the role your church leaders or fellow members of your church play in your diabetes management behavior?Probe: Is there something about the church environment that makes this comfortable or uncomfortable?Probe: Does your faith leader or other members of the church visit the sick to pray over them or offers words of support?
**Religious beliefs in the health setting**
9. What benefit, if any, is there in discussing religious beliefs or practices with your doctor, nutritionist, nurse, or any other health professional? Are there negative things that can come from this discussion?Probe: What makes it comfortable or uncomfortable for you to talk about these things with your doctor?

A single, trained interviewer who was bilingual (English/Spanish) conducted one-on-one interviews. Each interview was audio-recorded and transcribed. Spanish language interviews were translated for the purpose of dissemination of results. Transcriptions were analyzed using Atlas.ti 8. Coding of each interview was completed in the order in which interviews were conducted, and an inductive coding approach was utilized. A phenomenological analytical approach was used to help understand the experience of managing diabetes through the lens of individuals in this culture, and to understand the utilization and/or application of religiosity and/or fatalism to help cope with the disease. An effort was made to complete interviews by respective Salud y Vida engagement levels to ensure that equal representation by group was achieved when determining saturation. Engagement was determined by the duration of participation in the program and participation in various programmatic elements. Saturation was deemed to be met after no additional codes were identified within an iteration of interviews by engagement level. Because each individual was interviewed once and because a phenomenological approach was used, it was expected that the sample would reach saturation after 10–15 interviews, which is consistent with prior research Creswell (22) and Morse (23). Given the research aim posed by the proposed study intended to examine participants' beliefs among three different levels of engagement, a total of 15 interviews were conducted to ensure saturation was met (24).

Excerpts were selected from transcripts to elaborate on identified themes. A secondary independent reviewer evaluated the selected codes and excerpts to ensure appropriateness in describing themes, and to reach a consensus in generating one list of themes. Authors noted the count of the occurrence of identified themes by engagement levels and gender. Disproportionality in the occurrence of themes was determined if a difference by at least two respondents expressing the belief was noted between groups.

### Participant Selection

The study population included participants enrolled in Salud y Vida, a chronic care management program for patients with T2DM. This ongoing program first opened its doors in South Texas in 2012, and offers an opportunity to explore the relationship between fatalism, religiosity, and T2DM management. Salud y Vida provides Diabetes Self-Management Education (DSME) classes, and quarterly, home visits from a community health worker (*promotora)*. Inclusion criteria for participants include being over 18 years of age, having uncontrolled diabetes (HbA1c over 8.0%), and residing within the Regional Healthcare Partnership region 5. Exclusion criteria include having a history of violent behavior, history of substance abuse, currently receiving dialysis, currently a cancer patient, having open chronic wounds, having untreated bipolar or personality disorders, or are currently pregnant.

To understand the manner in which fatalism and religiosity play a role in facilitating or hindering diabetes self-care accounting for exposure to programing provided by Salud y Vida, the study sample was stratified by newly, currently, and long-term enrolled participants. Newly enrolled is defined as having been an active participant in the program for 45 days or less, and not having had a first *promotora* home visit, or not having attended the Diabetes Self-Management Education (DSME) class. Currently enrolled participants are those who have been active in the program for 46–352 days. Long-term enrolled participants are those enrolled and still receiving program services for 353 days or more. Stratification method was employed to allow for the observation of the effect of Salud y Vida intervention elements, if any, on fatalistic beliefs or religiosity among participants with varying levels of exposure to the program.

## Results

**Figure 1:**
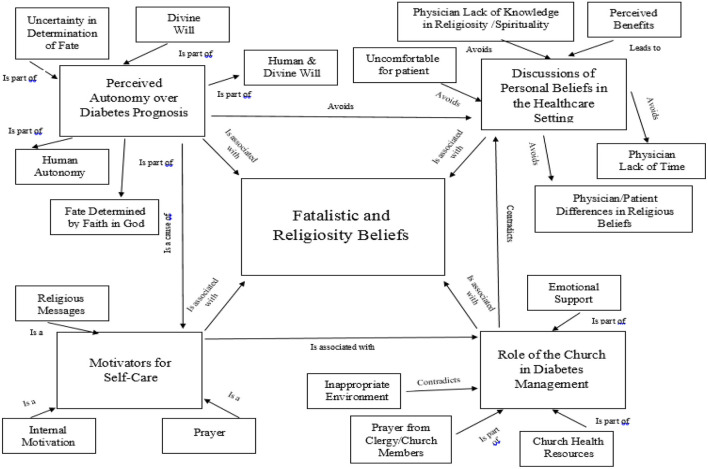
Theme network by level of engagement.

Interviews were conducted with 15 individuals between May to June, 2017. An equal number of respondents represented each level of engagement (5 newly, 5 currently, and 5 long-term enrolled). Average length of interviews was 24 min 59 sec (range: 13 min 10 sec−42min 12 sec). Participants were primarily female (73.3%), Spanish-speaking (66.7%), and the average age was 51.7 years old. Demographics by level of engagement can be found in Table 2. Major themes related to fatalism and religiosity and disease management included: (1) perceived autonomy over diabetes prognosis, (2) motivators for self-care, (3) discussions of personal beliefs in the healthcare setting; and (4) the role of the church in diabetes management. A complete list of themes, sub-themes, and their occurrence by gender and level of engagement can be found in Table 3. Figure 1 shows the network view of themes and sub-themes.

**Table 2:** Demographics by levels of engagement.

**Demographic factor**	**Newly enrolled**	**Currently enrolled**	**Long-term enrolled**
Gender			
Male	2	0	3
Female	3	5	4
Language preference			
English	2	1	2
Spanish	3	4	3
Average Age (years)	49.2 (12.8)	56.4 (20.45)	49.4 (15.8)

**Table 3:** Themes related to the role of religiosity and fatalism in Type 2 diabetes management.

**Theme/sub-theme**	**Newly enrolled**	**Currently enrolled**	**Long-term enrolled**	**Male**	**Female**
Perceived autonomy over diabetes prognosis					
• Human autonomy	X		X		X
• Divine will	X	X	X	X	X
• Human and divine control	X	X	X	X	X
• Fate determined by faith in God	X			X	X
• Uncertainty in whom or what determines fate	X	X		X	X
Motivators for self-care					
• Religious messages	X	X	X	X	X
• Internal motivation	X	X	X	X	X
• Prayer	X	X	X	X	X
Discussions of personal beliefs in the healthcare setting					
• Physician/patient difference in religious beliefs	X	X		X	X
• Physician lack of time	X	X			X
• Uncomfortable for Patient	X	X		X	X
• Physician lack of knowledge in religiosity/spirituality	X	X	X	X	X
• Perceived benefit	X	X	X	X	X
Role of the church in diabetes management					
• Emotional support		X	X	X	X
• Church health resources			X		X
• Prayer from clergy/church members	X	X		X	X
• Inappropriate environment	X		X	X	X

### Perceived Autonomy Over Diabetes Prognosis

Participants expressed a complex understanding of the interplay between human and divine will in the determination of diabetes and resultant complications. Differing understandings of fate varied between attributing a person's health to human will, divine will, or a combination of the two. A variation of these beliefs presented by participants was the belief that one's health is dependent on the level of intensity of their religious faith, and others expressed uncertainty in identifying who or what decides a person's fate. Female newly and long-term enrolled participants expressed an exclusive human autonomy over health while none of the currently enrolled or male participants expressed this belief.

Both genders and all engagement levels (newly, currently and long-term enrolled) expressed feeling their diabetes prognosis was divine will. One female, current participant shared, “*With diabetes we can make all the healthy changes but if our destiny is to die of diabetes then we will die of diabetes.”* This type of perception can make an individual feel powerless when managing their illness, and may result in poorer self-care behaviors. Another female, currently enrolled participant stated, “*God has given me life, and he will take it when he wants…That is my belief, and my faith in God*.” Although this participant showed resolve and peace in her faith, these beliefs can hinder an individual from seeking to adopt healthy behaviors.

In the same manner, both genders and all engagement levels expressed a joint locus of control of health. “*I do think God has a play in someone's destiny. But there [are] still things we can do. If we make changes in our life to help our health then we can change our destiny*.” Participants with a viewpoint of a joint locus of control seemed to be able to mitigate feelings of anxiety about self-management, and seemed to be optimistic about their disease management.

A variation of these perspectives of divine control and joint locus of control is the belief that one's fate is determined by one's commitment to God, level of faith, piety, or righteousness. This belief was only shared by male and female newly enrolled participants. A female participant shared, “*If we believe in God, we will have good health. Also, if we do good, then we will have everything*.” Although this participant expressed optimism in the notion that God rewards devout faith and good behavior, beliefs such as this can translate into a person feeling religious guilt or accepting diabetes complications as divine punishment.

On the other hand, both female and male newly and currently enrolled participants shared uncertainty in fully understanding the existence or determination of fate. A currently enrolled, female participant shared: “*At times I believe in fate and at times I don't. I feel I am just really at the wrong place at the wrong time. I feel it may be coincidence*.” However, those expressing uncertainty all noted a combination of human and divine will. This same participant later stated, “*I think God is responsible for our destiny. I think we can change our future though, because future is a bit different… I think*.”

When looking at differences by levels of engagement and gender, a larger proportion of long-term enrolled participants attributed health outcomes to a higher power with three of the five sharing that belief vs. only one newly enrolled and one currently enrolled participant. All of the men in the current sample identified God or a combination of God and humans in control of their health, rather than attributing health to human will only.

### Motivators for Self-Care

Identified motivators for self-care included religious messages, prayer, and internal motivation. Female and male participants at all engagement levels expressed that their religious engagement helped support positive health behaviors through scriptures that proscribed caring for one's physical health. One male, long-term enrolled participant shared: “*I mean the reason I am taking care of myself is because I have a love for my heavenly father. It is because I take care of myself that I can go out and do his will*.” Participants expressing this belief viewed their diabetes management positively, and in accordance with their faith.

Again, male and female participants at all engagement levels expressed prayer was a coping mechanism and a source of support to engage in positive health behaviors. A female, currently enrolled participant said, “*We just pray for His help to get better. Sometimes [prayer] does not heal the body, but it heals the mind*.” Participants mainly mentioned prayer and their relationship with God as a form of inspiration to engage in healthy self-care behaviors. This same participant later shared that having courage and support makes managing her diabetes “*not so bad*.” Although participants identified prayer a resource in their diabetes management, none of the participants stated that they would use prayer in lieu of medical care.

However, there were male and female participants at all engagement levels who saw no connection with religiosity and health behaviors, but rather drew from secularly driven, self-motivation to engage in positive health behavior. A female, long-term enrolled participant shared: “*Religion does not affect my Type 2 management at all. I don't drink or smoke, but it is not because of my religion. I would still not do it even if I didn't go to church. I have not heard any religious messages about health either*.” Participants who did not use prayer for health reasons did not express negative viewpoints of the church nor did they identify barriers to self-care that differed from participants who drew motivation from their religiosity. In the same light, none of the participants expressed that any religious messages or time spent in prayer were contraindicative or a barrier to self-care. There was no disproportionately noted in the occurrence of the belief that there was no connection between religiosity and health behaviors across groups.

### Barriers to Discussing Personal Beliefs in the Healthcare Setting

Barriers to discussing fatalistic and/or religious beliefs with physicians noted by participants were differences in religious beliefs, lack of time during a medical consultation, lack of knowledge on the subject on behalf of the physician, and lack of comfort in discussing such beliefs in that setting. Male and female newly and currently enrolled participants expressed differences in religious beliefs as a barrier. A male, newly enrolled participant shared, “*I don't think I would ask a doctor on that part. He could bear a different religion than I have. It's like with different politics in Democrat or Republican*.” This sentiment could reflect a perception of lack of cultural competence on behalf of the physician.

Participants of both genders and all engagement levels felt that physicians did not have knowledge in the subjects of religion and spirituality. A male, newly enrolled participant shared, “*They don't have expertise. He is there to help me out physically… not as much emotionally or tell me about different religions.that comes from someone else like a clergy person…*.” Participants sharing these viewpoints all referenced their doctors as males, and mentioned a distinct disconnect between their physician and their personal beliefs. None of the participants spoke of their physician in a negative way; however, feelings of disconnect such as this may be due to participants not having experienced a physician inquire about their personal beliefs.

There were patients, however, of both genders and all engagement levels that felt it would be beneficial to discuss religious or fatalistic beliefs with health practitioners. One female, currently enrolled participant stated, “*I wish more doctors would pray. I think it would put a lot of people at ease*.” Participants believing a medical consultation was an appropriate setting for these discussions appeared to want “more” of their consultations. A female, currently enrolled participant shared, “*I think it would be good to talk to a doctor about this stuff. It would make the approach feel more ‘human', and it would be more beneficial. At times people feel inundated with problems concerning their disease…*” These participants may be feeling over-burdened by their self-management regimen, or may be worried about the prognosis of their disease.

When observing differences by gender and engagement level, none of the males or long-term enrolled participants cited lack of time as a barrier. Long-term enrolled participants as well did not reference differences in religious beliefs, or feelings of discomfort as barriers to these discussions. Individuals who expressed religious beliefs and practices playing a role in their understanding and management of diabetes felt discussing these beliefs with their physician would be beneficial. However, they felt that their physicians may not have the expertise needed to engage in these discussions.

### Role of the Church in Diabetes Management

Male and female current and long-term enrolled participants expressed that church members were a source of emotional support in dealing with their diabetes. A female, currently enrolled participant shared, “*So every Wednesday at 7 o'clock we have a group of friends…we have the rosary and then we get together like a therapy group…We discuss our problems. It is a therapy session for me*.” Only one female, long-term enrolled participant stated that her church had health resources. “*At the church that I go to they do give classes on health*. [The pastor] *talks to us about how we need to take care of our health and eat a healthy diet to care for our illnesses and to prevent them*.” None of the participants, noting any type of support or advice offered through the church, utilized these resources in lieu of medical care nor was it contradictory to the advice given to them by medical professionals.

Male and female newly and currently enrolled participants pointed to intercessory prayer by faith leaders and members of the congregation. A male, newly enrolled participant shared “*I am very fortunate that the pastors at my church…will go to your house and pray for you. When they have an alter call you can go and ask that you need strength or wisdom on your diabetes…*” Among this sample, those who engaged in intercessory prayer for others or were on the receiving end of intercessory prayer attributed emotional and social benefits to prayer.

On the other hand, one female, long-term enrolled participant and one male, newly enrolled participant did not feel that the church was the appropriate environment to discuss their diabetes care or management. The male participant shared, “*I think it just makes me uncomfortable because a church is [a] place to go in and praise God …I don't think people want to hear about your diabetic problem. In this region…there is so much diabetes that it is not even an interesting topic anymore*.” This individual spoke fondly of his church, and seemed to benefit from prayer by church leaders. However, this individual did not feel comfortable talking about his struggles with church members. Responses for this theme did not vary by gender; however, none of the newly enrolled participants felt that the church played a role in their self-management other than through intercessory prayer.

## Conclusions

Study findings exhibit a complex interplay of religiosity and fatalistic beliefs in an individual's comprehension of their disease and the way they approach its management. Among this sample, long-term enrolled participants and males were more fatalistic and believed religious beliefs and practices played a role in their diabetes management. Previous research examining diabetes management among African American and Hispanic populations have found perceived autonomy over disease outcomes to be a barrier for both men and women (25–27).

Beliefs in a divine will over health have been noted among non-Hispanic white and African American populations; however, these fatalistic beliefs were associated with higher HbA1c values, and poorer diet, exercise, and blood sugar monitoring behaviors (28). Similarly, among Jewish patients with T2DM there was an association between diabetes fatalism and a higher HbA1c; however, this relationship disappeared after controlling for religiosity (29). In the current study, long-term participants, who remain in the program because they have not succeeded in lowering their HbA1c to a point where they can be discharged, also share these beliefs, underscoring the role these beliefs play in self-care and their connections to distress in managing the disease. On the other hand, feelings of either joint human and divine autonomy or exclusive human autonomy over one's self-management, such as were noted among participants in all engagement levels and genders, can result in better adherence to self-care behaviors (30).

Looking at motivators for self-care, the link between religiosity and health behaviors through the prescriptive and proscriptive nature of religious messages is noted in a variety of religions (15). Participants sharing this viewpoint had a positive outlook on their self-care, which suggests that religious beliefs and teachings may be an important resource to overcome fatalistic beliefs. The use of prayer over health has been observed in previous research among Hispanic populations (17, 31). A national survey among American adults assessing prayer and spiritual practices reported 49.5% of Hispanic adults utilized prayer for their personal health and the health of others (31). A qualitative study among Hispanic farmworkers also found prayer to be utilized for diabetes healing and management (17). Findings of these studies and the present study suggest that religious beliefs and practices, namely prayer, may be an ancillary agent to addressing methods and strategies to improving blood glucose management among Hispanic populations.

Although participants listed barriers to discussing fatalistic and religious beliefs with their physicians, participants across all engagement levels and genders expressed a desire to openly discuss these beliefs in the context of their diabetes management. However, given that participants felt that physicians may not have the time or expertise to address these beliefs, it may be more suitable for these beliefs to be addressed by other health professionals, such as nurses or health educators. Moreover, it is important that in order for these discussions to be implemented that a strong interpersonal relationship be developed, and that providers approach these discussions in a culturally sensitive manner. A focus group conducted with women coping with breast cancer found that discussions regarding religiosity were only appropriate in instances where this type of interpersonal relationship existed (32). Participants expressing an unwillingness to take up time within their medical consultation to discuss these beliefs is similar to the findings of a cross-sectional study among out-patient medical clinics in that only 10% of the surveyed sample were willing to trade off time during their consultation to discuss religious beliefs (33). Nonetheless, discussions of these beliefs with a physician or other health professional are associated with higher patient satisfaction (34).

Participants' beliefs concerning the church's role in addressing T2DM as well reflect a desire for interpersonal connections in the context of health and exhibit the benefit of faith-based or faith-placed health interventions. Studies have shown that social and emotional support in the church setting may be protective of health and even more of a significant predictor of health than intrinsic religiosity (35, 36). More importantly, health messages delivered within the church setting may be the bridge needed for individuals to address their physical health as these messages are being delivered in a familiar setting by a trusted individual (37). Studies assessing the effects and/or benefits of intercessory prayer have shown mixed results (38, 39). Nevertheless, intercessory prayer has been noted as a coping mechanism among minority populations (40, 41).

Exploration of both religiosity and fatalism among participants is a strength of the present study. Although distinct, fatalism and religiosity are intertwined for some in their understanding of their health and wellness. Another strength of the current study is the representation of varying levels of engagement in the Salud y Vida program. This diverse representation allowed for the observation in differing themes by these groups. Limitations of the study are that all of the participants had uncontrolled diabetes; thus, viewpoints of individuals who are effectively managing their diabetes are not reflected. Another limitation is that stratification did not account for the number of years an individual had T2DM. Although gender differences were observed, stratification methods did not account for gender either.

Study findings underscore a need to address fatalism and religiosity in discussions within intervention programs addressing diabetes self-management behaviors. Discussions of these beliefs in a more familiar setting, such as a diabetes management class, with a community health worker, or a nurse in the clinic setting may overcome barriers identified to discussing fatalism and religiosity with a physician. Campos provides a variety of beliefs, such as *fatalismo*, that were noted among this sample and should be accounted and/or addressed when providing treatment for Hispanic patients with T2DM (42). Other concepts listed are *personalismo*, which pertains to the need for a perceived warm, personal patient-provider relationship, and *respecto*, which refers to respect offered based on social standing, age, and/or gender. Although Campos discusses these concepts through the lens of a physician and patient relationship, findings of this study suggest a need to address these beliefs in alternative manners in the healthcare setting (42).

Findings as well suggest a need for assessment of religious beliefs prior to consultation or treatment among this population. Lujan and Campbell (43) note the importance of assessing such beliefs to ensure practitioners provide holistic treatment and education. In assessing religious beliefs, it is also important to note one's own biases and beliefs to provide objective treatment. These assessments provide educators with the understanding to identify barriers to self-care and appropriate opportunities to refer to faith leaders in the patient's treatment if necessary (43).

Discussions surrounding fatalistic beliefs among this sample point to a generally positive outlook on self-agency in diabetes management. Additionally, many participants drew positive support from their church and religious teachings and practices for their self-care. Although fatalistic beliefs are negatively associated with social and psychological well-being among Hispanics, they are positively associated with general well-being (44). Seeing the complex understandings participants expressed of fate and its role in diabetes management, it is important to address these beliefs in diabetes education to increase patient self-efficacy in management care behaviors. While this study demonstrated new understanding in the manner in which fatalistic and religiosity beliefs may play a role in an individual's understanding and management of T2DM, future research should explore this relationship while controlling for other demographic factors, such as health literacy levels, acculturation, and length of time coping with T2DM. Moreover, future research should aim to explore these thoughts and beliefs among a more varied sample, such as comparing individuals with HbA1c levels that represent good vs. poor glycemic control, comparing different religious beliefs/backgrounds, and/or including individuals with varying levels of diabetes complications. The current study is limited to individuals with HbA1c levels above 8.0%, a predominantly religious convenience sample, and excludes individuals undergoing kidney dialysis. Future research should as well aim to assess how fatalistic and religious beliefs affect individuals' lived experiences with type 2 diabetes to gain further insight as to how these beliefs influence an individual's perception of care beyond their health behaviors as assessed in the current study.

## Data Availability Statement

The raw data supporting the conclusions of this article will be made available by the authors, without undue reservation.

## Ethics Statement

The studies involving human participants were reviewed and approved by University of Texas Health Science Center at Houston School of Public Health. The patients/participants provided their written informed consent to participate in this study.

## Author Contributions

CS-C conducted, transcribed and analyzed the semi-structured interviews. BR served as the secondary coder during the data analysis process. All authors contributed to the development of the present study's data collection tools and methodologies, writing, and editing of the final manuscript.

## Conflict of Interest

The authors declare that the research was conducted in the absence of any commercial or financial relationships that could be construed as a potential conflict of interest.

## Publisher's Note

All claims expressed in this article are solely those of the authors and do not necessarily represent those of their affiliated organizations, or those of the publisher, the editors and the reviewers. Any product that may be evaluated in this article, or claim that may be made by its manufacturer, is not guaranteed or endorsed by the publisher.
